# BMC Urology reviewer acknowledgement, 2012

**DOI:** 10.1186/1471-2490-13-15

**Published:** 2013-04-23

**Authors:** Hayley Henderson

**Affiliations:** 1 BioMed Central, Floor 6, 236 Gray's Inn Road, London, WC1X 8HB, United Kingdom

## Abstract

**Contributing reviewers:**

The editors of *BMC Urology* would like to thank all our reviewers who have contributed to the journal in Volume 12 (2012).

## 

David Aaronson

USA

Piyush Agarwal

USA

Neena Agarwala

USA

Sarfraz Ahmad

United Kingdom

Hamed Akhavizadegan

Iran

Mehrdad Alemozaffar

USA

Satoshi Anai

Japan

Mohammad Asim

United Kingdom

Lionel Banez

USA

Jim Barber

United Kingdom

Selahattin Bedir

Turkey

Francesco Berardinelli

Italy

Bart Biallosterski

Netherlands

Tina Bianco-Miotto

Australia

Dale Bjorling

USA

Peter Black

Canada

Yanis Boumber

USA

Benjamin Breyer

USA

Matteo Brunelli

Italy

David Buethe

USA

Karl Chai

USA

Christopher Chapple

United Kingdom

Christopher Chermansky

USA

Luca Cindolo

Italy

Julia Crook

USA

Giorgia De Berardis

Italy

Antonina Di Benedetto

Italy

Rian Dickstein

USA

Thorsten H. Ecke

Germany

Nabil El Aila

Belgium

Ahmed El-Sakka

Saudi Arabia

Cesar E Ercole

USA

Aude Flechon

France

Jay Fowke

USA

Nancy Fultz

USA

Maurice Garcia

USA

Holger Gerullis

Germany

Evan Gomes

USA

Marco Grande

Italy

Alaa Hamada

USA

Hannelore Heemers

USA

David Hernandez

USA

Warren Hill

USA

Robert Hurst

USA

Kenneth Iczkowski

USA

Kubilay Inci

Turkey

Debra E. Irwin

USA

Gianmarco Isgro'

Italy

Jonathan Izawa

United Kingdom

Wassim Kassouf

USA

Toshihiko Kawamori

USA

Amir Kazzazi

USA

Carsten Kempkensteffen

Germany

Kittinut Kijvikai

Thailand

Hari Koul

USA

Keith Kowalczyk

USA

Hann-Chorng Kuo

Taiwan

Patrick Kupelian

USA

Masaomi Kuwada

Japan

Fanuel Lampiao

Malawi

Dirk Lange

Canada

Brett Lebed

USA

Costantino Leonardo

Italy

David Levy

USA

Evangelos Liatsikos

Greece

Hsueh-Kung Lin

USA

Conor Lynch

USA

Reda Mahfouz

USA

Ahmed Mansour

USA

Alayne Markland

USA

Angabin Matin

USA

Lourdes Mengual

Spain

Adam Metwalli

USA

Majid Mirzazadeh

USA

Gayatri Mohanty

India

Jim Mossman

USA

Atsushi Nagai

Japan

Yasushi Nakai

Japan

Kazunori Namiki

Japan

Antonio Nouvenne

Italy

Rahmi Onur

Turkey

Blake Palmer

USA

Chin-Chen Pan

Taiwan

Liliana Pascual

Argentina

Lucrezia Pignatti

Italy

Nicholas Power

Canada

Jenny Poynter

USA

Sanoj Punnen

USA

Victoria Raymond

USA

Alice Rickard

USA

Wasia Rizwani

USA

Ryuji Sakakibara

Japan

Aruna Sarma

USA

Wolfgang Schulz

Germany

Stephen Schwartz

USA

Wade Sexton

USA

Katsumi Shigemura

Japan

Brian Shuch

USA

Praveen Singam

Malaysia

Eric Singer

USA

Armine Smith

USA

Mark Soloway

USA

Carole Sourbier

USA

Philippe Spiess

USA

Sushma Srikrishna

United Kingdom

Lambros Stamatakis

USA

Kimio Sugaya

Japan

Debasish Sundi

USA

Martin Sundqvist

Sweden

Robert Svatek

USA

Shin-Ichi Takeda

Japan

David Thiel

USA

Jeffrey Triest

USA

Norihiko Tsuchiya

Japan

Virginia Urquidi

USA

Bahareh Vahabi

United Kingdom

Adrie Van Bokhoven

USA

Alex Varghese

USA

Gan Wang

USA

Sean Williamson

USA

Yue Wu

USA

Takahiro Yasui

Japan

Stanley Zaslau

USA

Francesco Ziglioli

Italy

Ellen Zwarthoff

Netherlands

## Pre-publication history

The pre-publication history for this paper can be accessed here:

http://www.biomedcentral.com/1471-2490/13/15/prepub

